# Key Amino Acid Residues That Determine the Antigenic Properties of Highly Pathogenic H5 Influenza Viruses Bearing the Clade 2.3.4.4 Hemagglutinin Gene

**DOI:** 10.3390/v15112249

**Published:** 2023-11-13

**Authors:** Yuancheng Zhang, Pengfei Cui, Jianzhong Shi, Yuan Chen, Xianying Zeng, Yongping Jiang, Guobin Tian, Chengjun Li, Hualan Chen, Huihui Kong, Guohua Deng

**Affiliations:** 1 State Key Laboratory of Animal Disease Control and Prevention, Harbin Veterinary Research Institute, Chinese Academy of Agricultural Sciences, Harbin 150009, China; 18374874726@163.com (Y.Z.); cuipengfei@caas.cn (P.C.); shijianzhong@caas.cn (J.S.); charlesyg1@163.com (Y.C.); zengxianying@caas.cn (X.Z.); jiangyongping@caas.cn (Y.J.); tianguobin@caas.cn (G.T.); lichengjun@caas.cn (C.L.); chenhualan@caas.cn (H.C.); 2 Guangdong Laboratory for Lingnan Modern Agriculture, Guangzhou 510642, China

**Keywords:** antigenicity, H5, clade 2.3.4.4, glycosylation, cartography

## Abstract

The H5 subtype highly pathogenic avian influenza viruses bearing the clade 2.3.4.4 HA gene have been pervasive among domestic poultry and wild birds worldwide since 2014, presenting substantial risks to human and animal health. Continued circulation of clade 2.3.4.4 viruses has resulted in the emergence of eight subclades (2.3.4.4a–h) and multiple distinct antigenic groups. However, the key antigenic substitutions responsible for the antigenic change of these viruses remain unknown. In this study, we analyzed the HA gene sequences of 5713 clade 2.3.4.4 viruses obtained from a public database and found that 23 amino acid residues were highly variable among these strains. We then generated a series of single-amino-acid mutants based on the H5-Re8 (a vaccine seed virus) background and tested their reactivity with a panel of eight monoclonal antibodies (mAbs). Six mutants bearing amino acid substitutions at positions 120, 126, 141, 156, 185, or 189 (H5 numbering) led to reduced or lost reactivity to these mAbs. Further antigenic cartography analysis revealed that the amino acid residues at positions 126, 156, and 189 acted as immunodominant epitopes of H5 viruses. Collectively, our findings offer valuable guidance for the surveillance and early detection of emerging antigenic variants.

## 1. Introduction

Influenza A viruses are categorized based on the antigenic or genetic diversity of their surface proteins, hemagglutinin (HA) and neuraminidase (NA). To date, 18 HA and 11 NA subtypes have been identified in their natural hosts. Among these, 16 HA subtypes (H1–H16) and 9 NA subtypes (N1–N9) were isolated from wild aquatic birds, whereas the H17N10 and H18N11 subtypes were detected in bats [1,2]. The H5 subtype stands out as one of the only two subtypes capable of evolving into highly pathogenic avian influenza viruses (HPAIVs) [3,4,5,6,7,8,9,10]. These H5 HPAIVs have caused considerable economic losses in the global poultry industry [4]. Beyond their impact on poultry, H5 HPAIVs also represent a potential pandemic risk. As of 22 September 2023, H5 viruses have caused 965 human infections, resulting in 491 fatalities worldwide since January 2003 [11]. Given this threat, it is imperative to strengthen control strategies, such as enhanced surveillance and proactive vaccination of poultry.

H5 HPAIVs have evolved into 10 primary genetic clades (clades 0–9) and multiple subclades [12]. Currently, the dominant H5 viruses circulating worldwide are clade 2.3.4.4 viruses, which have formed eight subclades (clade 2.3.4.4a–h) and multiple antigenic groups [13,14,15,16,17]. Early in 2014, novel H5N8 reassortant viruses with HA genes derived from H5N1 viruses bearing the clade 2.3.4.4 HA gene were identified in South Korea [18]. These viruses then spread to Europe, North America, and East Asia, largely driven by migratory bird movements [19]. In response to the threats posed by the clade 2.3.4.4 H5 viruses, the H5-Re8 vaccine strain classified under the 2.3.4.4g clade in accordance with the WHO nomenclature system [20] was introduced. This vaccine effectively curtailed the proliferation of clade 2.3.4.4g viruses among poultry in China. Yet, in 2017, the emergence of the antigenically distinct clade 2.3.4.4h viruses in Chinese poultry led to a transition from the H5-Re8 vaccine strain to H5-Re11 [14]. In 2020, a novel H5N8 virus carrying the clade 2.3.4.4b HA caused outbreaks in Polish chickens and subsequently triggered outbreaks across poultry and wild birds in Europe, Africa, and Asia [4,19,21,22]. Concurrent with the isolation of the new antigenically unique clade 2.3.4.4h viruses, the Chinese government transitioned to the clade 2.3.4.4 vaccine strains H5-Re13 and H5-Re14. These provided defenses against the emerging 2.3.4.4h and 2.3.4.4b viruses, respectively [15]. Although these vaccines successfully controlled these antigenically different clade 2.3.4.4 viruses [14,15,16,23], our understanding of the antigenic determinants of these viruses remains limited.

Numerous studies have shown that the major antigenic changes observed in the evolution of seasonal H3N2, clade 2.1 H5N1 viruses, as well as recent antigenic shifts in seasonal H1N1 and influenza B viruses, were predominantly driven by substitutions near the receptor binding site [24,25,26,27]. However, we lack a comprehensive understanding of the antigenicity of the currently circulating clade 2.3.4.4 H5 viruses. Specifically, the antigenic properties of each amino acid position in the HA of clade 2.3.4.4 have yet to be fully elucidated. To discern which amino acid substitutions might influence the antigenicity of these viruses, we reviewed all available HA sequences of H5 viruses and identified 23 potential amino acid positions for antigenic examination. By creating various mutants and assessing their antigenicity using both polyclonal and monoclonal antibodies, we pinpointed six primary antigenic positions: 120, 126, 141, 156, 185, and 189 (H5 numbering, used hereafter). Notably, three of these positions, namely 126, 156, and 189, appear to be dominant in eliciting antibody responses in hosts. Our findings provide insights into viral immune evasion mechanisms and offer guidance for vaccine strain selection.

## 2. Materials and Methods

### 2.1. Sequence Analysis

HA sequences of clade 2.3.4.4 H5 HPAIVs, collected from January 2013 to August 2023, were sourced from the GISAID database (https://gisaid.org/, accessed on 15 August 2023). After filtering out identical sequences, the antigenic regions of 5713 sequences, inferred from 3D structural comparisons of H5 HA and human H3 HA [28,29], were analyzed using Tbtools, as previously detailed [30,31].

### 2.2. Viruses and Cells

The H5 vaccine strain, H5-Re8, was derived from the surface genes of A/chicken/Guizhou/4/2013(H5N1) and the backbone of A/Puerto Rico/8/1934(H1N1) and was maintained in our lab. Mouse myeloma (Sp2/0) and human embryonic kidney (293T) cells were cultured in DMEM supplemented with 10% Fetal Bovine Serum (FBS) and cultured at 37 °C in a 5% CO_2_ environment.

### 2.3. Production and Purification of Monoclonal Antibodies

Using the hybridoma technique, we produced specific mAbs [32]. Female BALB/c mice, aged 6 weeks, were intramuscularly injected with 50 μg of the pCAGGS vector expressing the HA protein of H5-Re8. Fourteen days after the initial injection, these mice received two booster injections, each containing 10 µg of the inactivated H5-Re8 vaccine. By applying cell fusion methods, we generated hybrid cells by fusing Sp2/0 myeloma cells with antibody-secreting cells. The positive hybridoma cells were identified by using the hemagglutinin inhibition (HI) assay. Subsequently, monoclonal hybridoma cells (1.0 × 10^4^ per mouse) were intraperitoneally injected into BALB/c mice that had been sensitized with Freund’s incomplete adjuvant (500 μL per mouse). Antibodies (ascites) extracted from the mice were purified by using a protein G affinity column (GE Healthcare, Chicago, IL, USA), and the concentration of the purified mAbs was then determined by using a BCA protein assay kit (Beyotime, Shanghai China).

### 2.4. Construction of Recombinant Mutant Virus

Mutants were generated based on the H5-Re8 vaccine virus previously developed in our laboratory. Recombinant virus rescue employed a reverse genetic approach as previously described [32]. In brief, 293T cells were seeded in 6-well plates a day before transfection. Adhering to the prescribed protocol provided by the manufacturer, the 293T cells were transfected with 4 μg of a mixture containing eight distinct plasmids (0.5 μg each) using the transfection agent Lipofectamine^®^ 3000 (Invitrogen, Waltham, MA, USA). At 8 h post-transfection, the culture medium was substituted with Opti-MEM and supplemented with 0.125 μg/mL of TPCK-trypsin (Sigma, St. Louis, MO, USA). The cells were then incubated at 37 °C under 5% CO_2_ for 48 h. Then, the supernatant from the transfected cells was collected and used to inoculate ten-day-old embryonated chicken eggs. Following incubation at 37 °C for an additional 48 h, the allantoic fluid was harvested, and the presence of introduced mutations, as well as the absence of any unwanted mutations, was confirmed through Sanger sequencing.

### 2.5. Hemagglutination Inhibition and Microneutralization Assays

Hemagglutination inhibition (HI) assays were performed as previously described [3], using a set of immunizing chicken antisera or mAbs. Each serum or mAb was mixed with 25 μL of PBS containing four hemagglutinating units of the specific virus. After a 20 min incubation at ambient temperature, 25 μL of 1% chicken erythrocytes was added. Following a subsequent 20 min incubation, inhibition titers were determined as the inverse of the highest serum dilution required to fully prevent chicken erythrocyte agglutination.

The microneutralization (MN) assay was conducted based on previously established methods [33]. First, the serum samples underwent a 2-fold serial dilution with DMEM containing 1 μg/mL TPCK-trypsin, and then 50 μL of the diluted serum was mixed with 50 μL of DMEM containing 100 TCID_50_ (50% tissue culture infectious dose) of virus. After a 1 h incubation at 37 °C, the mixture was dispensed onto MDCK cells in 96-well plates. The microneutralization titer corresponds to the peak dilution that effectively suppresses viral replication.

### 2.6. Antigenic Cartography

Using the online tool at https://www.antigenic-cartography.org/, accessed on 10 October 2023, we assessed the antigenic distances between viruses, as previously described [34].

## 3. Results

### 3.1. Inferring Antigenic Positions in the HA of Highly Pathogenic Clade 2.3.4.4 H5 Viruses

**Figure 1:**
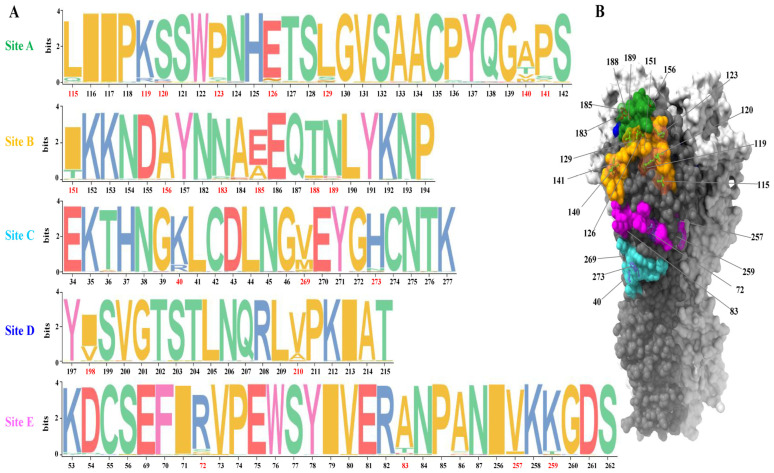
Amino acid polymorphism at five antigenic regions of clade 2.3.4.4 viruses and their relative locations on the globular head domain of HA. (**A**) Antigenic regions of H5 HA were inferred by mapping the three-dimensional structure of H5 HA (PDB accession number, 5HUF) onto the 3D structure of H3 HA (PDB accession number, 4WE4). Amino acid polymorphism in these regions was analyzed using TBtools. (**B**) The relative locations of the putative antigenic positions are highlighted on the 3D structure of H5 HA.

The evasion of influenza A viruses from the host immune system is mainly mediated through its surface protein HA. During the past decade, the clade 2.3.4.4 viruses have undergone extensive genetic and antigenic evolution, resulting in the formation of eight subclades (clade 2.3.4.4a–h). Antigenic variations have been observed between different clades and even within the same clade [14,15,16,21]. However, the specific antigenic epitopes driving the antigenic diversity of clade 2.3.4.4 viruses remain unclear. To gain a comprehensive understanding of the antigenic epitopes of H5 viruses carrying clade 2.3.4.4 HA, we mapped the 3D structure of H5 HA to the five antigenic epitope regions (A–E) of human H3N2 viruses [28,29] (Figure 1B). By analyzing a total of 5713 HA sequences downloaded from the database using a method previously described [31,35], we inferred that antigenic region A exhibited the highest frequency of amino acid change, with eight potential antigenic positions (i.e., 115, 119, 120, 123, 126, 129, 140, and 141, Figure 1A). For antigenic region B, six possible antigenic positions (151, 156, 183, 185, 188, and 189) were identified, and for antigenic region C, three potential antigenic positions (40, 269, and 273) were identified. At antigenic region D, there were two possible antigenic positions (198 and 210), and at antigenic region E, there were four potential antigenic positions (72, 83, 257, and 259). In summary, a thorough analysis of amino acid polymorphisms across the five antigenic regions identified 22 potential antigenic positions.

### 3.2. Rescue Reassortant Mutants with a Single Amino Acid Substitution

To ascertain which position might determine the antigenicity of H5 viruses, we generated a series of mutants (Table 1), each with a single amino acid substitution, using reverse genetics based on the vaccine strain H5-Re8 [36]. This study probed a total of 23 potential antigenic positions, which included the 22 positions inferred from the sequence analysis and position 156, which has been shown to influence the formation of a glycosylation site and the antigenicity of influenza viruses in other studies [37,38,39]. It is noteworthy that some HA sequences contain NHΔT (Δ indicates an amino acid deletion) at positions 124–127 (Figure 1), whereas H5-Re8 lacks a glycosylation motif at the corresponding positions, with DHDT at positions 124–127. Therefore, a D124N mutation was introduced, accompanied by an amino acid deletion at position 126, to create a glycosylation site at positions 124–127 (DHDT to NHΔT) (Figure 1 and Table 1). All mutants were successfully rescued and exhibited growth titers in eggs similar to those of the H5-Re8 vaccine strain. The hemagglutination titers (HAU) varied from 7 log2 to 9 log2. These results imply that H5 HA possesses sufficient flexibility to accommodate single amino acid substitutions at these positions, suggesting that these individual mutations do not impair the functionality of HA.

### 3.3. Confirmation of Glycosylation at Antigenic Positions

**Figure 2:**
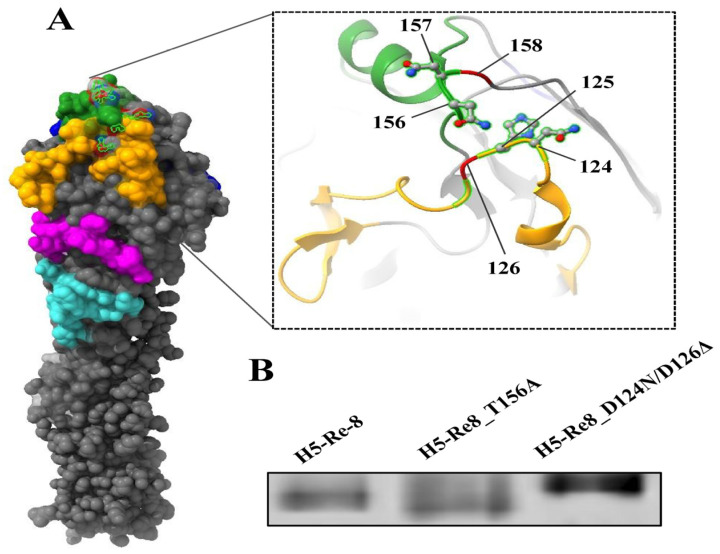
The location and confirmation of the glycosylation sites of HA protein. (**A**) Glycosylation sites were mapped onto the 3D structure of H5 HA (PDB accession number, 5HUF). (**B**) Diverse HA mobility of the mutants H5-Re8_D124N/D126Δ and H5-Re8_T156A in comparison with H5-Re8 in Western blot analysis.

While the presence of an N-X-T/S motif in a protein sequence provides a straightforward method to predict N-linked glycosylation, it is important to recognize that not all motifs result in glycosylation [40]. To verify whether D124N/D126Δ and T156Ainduced mutations at potential glycosylation sites indeed affected N-linked glycosylation at residues 124N and 154N, respectively, the HA of the mutants H5-Re8_D124N/D126Δ and H5-Re8_T156A were analyzed by Western blotting. Virus lysates were analyzed for HA mobility and changes in the size of the HA bands on Western blotting were observed. The size difference suggests a change in the size of the expressed HA, consistent with the addition (for H5-Re8_D124N/D126Δ) or deletion (for H5-Re8_T156A) of glycosylation sites (Figure 2). 

### 3.4. Mapping of Antigenic Positions on H5 HA by Using Monoclonal Antibodies

**Figure 3:**
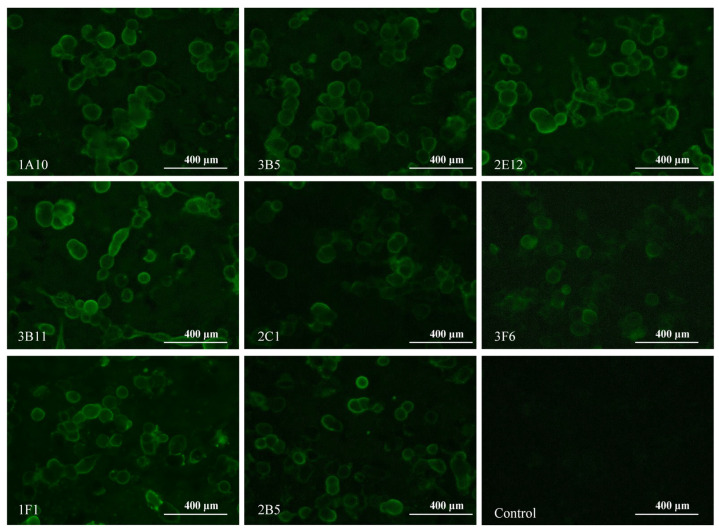
Reactivity of mAbs with H5-Re8 HA protein as detected via the use of an indirect immunofluorescence assay. The 293T cells were transfected with a plasmid expressing the H5-Re8 HA protein, and mAbs (concentration 40 μg/mL) were used to detect H5-Re8 HA expression.

Monoclonal antibodies (mAbs), a crucial tool for distinguishing subtle differences between antigens, have been extensively employed to map antigenic epitopes of HA proteins in various studies [41,42,43,44,45]. To elucidate the antigenic epitopes of H5-HA, we generated a panel of eight mAbs by immunizing mice with H5-Re8 HA. The successful selection of hybridoma cell lines was validated using the hemagglutination inhibition assay (HI). As shown in Table 2, the eight mAbs, designated as 2C1, 1A10, 2E12, 2B5, 3F6, 3B11, 1F1, and 3B5, effectively inhibited the interaction between the H5-Re8 virus and chicken erythrocytes, exhibiting HI titers ranging from 128 to 256. We first tested the specificity of the eight mAbs to H5-Re8 HA by performing an indirect immunofluorescence assay (IFA) in cells transfected with a plasmid expressing the H5-Re8 HA protein. All eight mAb-treated cells displayed enhanced fluorescence, whereas the mock-treated cells did not, indicating that the eight mAbs have a high binding affinity for H5-Re8 HA (Figure 3). We then determined the isotypes of the eight mAbs by using an isotyping test kit. The mAbs were categorized into three distinct IgG subclasses: 2C1 was classified as IgM; 1A10 and 2E12 were classified as IgG2a; and the remaining five mAbs (i.e., 2B5, 3F6, 3B11, 1F1, and 3B5) were classified as IgG2b (Table 2). All the mAbs possessed κ light chains. Lastly, we tested the effect of the eight mAbs on H5-Re8 replication by using a microneutralization assay (MN). The results showed that all eight mAbs neutralized H5-Re8 replication at a concentration of 40 µg/mL (Table 2). These results demonstrate that all eight mAbs specifically recognize H5-Re8 HA and have inhibitory activity against the H5-Re8 virus.

**Figure 4:**
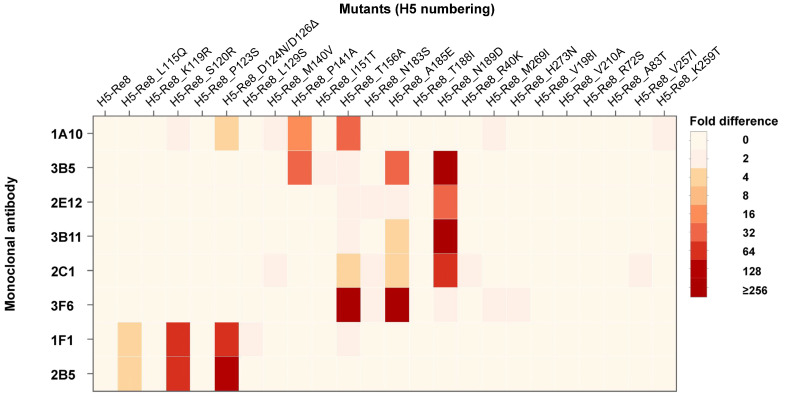
Antigenic heatmap of H5-Re8 mutants. The antigenicity of H5-Re8 variants was determined by quantifying hemagglutination inhibition (HI) titers using a panel of eight monoclonal antibodies (mAbs) developed in this study. An antigenicity difference is considered significant when there is a 4-fold difference.

To pinpoint the antigenic positions, the eight mAbs were cross-reacted with all mutants using the HI assay. As illustrated in the heat map generated from the HI titers (Figure 4), mAbs 1F1 and 2B5 displayed significantly low reactivity against H5-Re8_L115Q, H5-Re8_120R, and H5-Re8_D124N/D126Δ, with the lowest reactivity being exhibited against the H5-Re8_ D124N/D126Δ mutant. This finding suggests that mutations at these positions could influence H5-Re8 virus antigenicity, with mutations at the glycosylation site having a pronounced impact on the antigenicity of H5 viruses. Moreover, mAbs 3B5, 2E12, 3B11, and 2C1 demonstrated notably reduced reactivity against H5-Re8_N189D, with an antigenic difference ranging from 32- to 256-fold, compared with H5-Re8. Intriguingly, of these mAbs, 2E12 showed the most reduced reactivity only against H5-Re8_N189D. In contrast, 3B11 had reduced reactivity against the mutant H5-Re8_A185E, and 2C1 exhibited reduced reactivity against two other mutants, H5-Re8_T156A and H5-Re8_A185E. Similarly, 3B5 showed reduced reactivity against two mutants, H5-Re8_P141A and H5-Re8_A185E. For mAb 3F6, its reduced reactivity was most pronounced against the mutants H5-Re8_T156A and H5-Re8_A185E, aligning with the findings for 2C1. For mAb 1A10, it displayed the most reduced reactivity against H5-Re8_D126Δ, H5-Re8_P141A, and H5-Re8_T156A, with the mutation at the potential glycosylation site having the most profound effect on antigenicity. These data indicate that the identified antigenic positions may form conformational epitopes.

### 3.5. Mapping of the Antigenic Positions on H5 HA by Using Polyclonal Antisera

To assess the extent of the influence of each antigenic position on the antigenicity of H5 viruses, we assessed the antigenicity of the mutants by using polyclonal antisera generated from inactivated H5-Re8, H5-Re11, H5-Re13, and H5-Re14 vaccines, all of which are previous or current clade 2.3.4.4 vaccines [14,15]. The data revealed that, except for mutants H5-Re8_D124N/D126Δ and H5-Re8_T156A, all mutants exhibited no significant antigenic differences compared with the H5_Re8 virus. Specifically, the HI titer of H5-Re8_D124N/D126Δ against H5-Re8 antisera was 4-fold lower than that of H5-Re8, and the HI titer of H5-Re8_T156A against H5-Re13 antisera was 4-fold lower than that of H5-Re8 (Table 1). These findings underscore the key role of glycosylation in viral antigenicity and suggest that the residues at these positions may act as immunodominant epitopes.

### 3.6. Visualization of Antigenic Differences Using Antigenic Cartography

**Figure 5:**
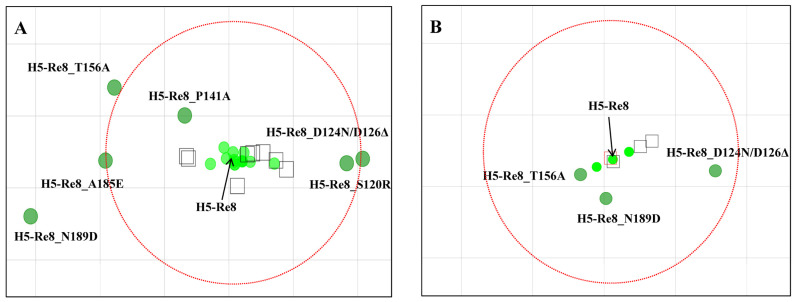
Antigenic cartography visualized with mAbs (**A**) and poly antisera (**B**). To more effectively depict the HI titers measured by mAbs and poly antisera, algorithms were used to interpret the HI titers. The red dashed circle represents a 4-fold antigenic difference from the H5-Re8 parent virus. Circles and rectangles signify viruses and sera, respectively. The red rectangle highlights homologous antisera against the H5-Re8 virus.

To better depict the antigenic differences among the mutants, HI titers were converted into an antigenic map using a previously described algorithm [34,46]. In the antigenic map constructed using data derived from the mAbs, four mutants—H5-Re8_D124N/D126Δ, H5-Re8_T156A, H5-Re8_A185E, and H5-Re8_N189D—showed significant antigenic differences from the H5-Re8 virus. H5-Re8_S120R and H5-Re8_P141A exhibited antigenic differences, although the differences were not significant (Figure 5A). Compared with the mAbs, the polyclonal antisera distinguished a limited number of antigenic sites (Figure 5B). Specifically, H5-Re8_D124N/D126Δ showed a notable antigenic difference from H5-Re8, whereas H5-Re8_T156A showed a less significant difference. Notably, although not identified with the polyclonal antisera, H5-Re8_N189D exhibited an antigenic difference from H5-Re8 in the map, but this difference was not significant. In summary, our data revealed that positions 120, 126, 141, 156, 185, and 189 are the primary antigenic positions for the clade 2.3.4.4 H5 viruses, with positions 126, 185, and 189 having a predominant impact on the antigenicity of H5 viruses.

## 4. Discussion

In this study, our sequence analysis inferred that the antigenic variation of clade 2.3.4.4 viruses may be caused by amino acid substitutions at 23 potential antigenic positions. Therefore, we systematically examined the impact of substitutions at these positions on the antigenicity of H5 viruses bearing clade 2.3.4.4 HA. We generated a panel of eight mAbs to discern subtle antigenic differences caused by point mutations. The data sourced from these mAbs identified six antigenic positions: 120, 126, 141, 156, 185, and 189. Mutations at positions 126 or 156 predominantly affected the antigenicity of H5 viruses, as determined by using polyclonal antisera. Intriguingly, the cartography map revealed that a substitution at an additional position (i.e., 189) also exerted a predominant role on the antigenicity of H5 viruses. These findings not only further our understanding of the molecular mechanisms of antigenic drift but will also aid in identifying potential epidemic strains, thereby assisting in the selection of vaccine candidates.

The HA protein of influenza A viruses exhibits high plasticity, allowing it to tolerate various mutations [47,48]. Peng et al. suggested that a total of 158 positions influence the antigenicity of H5 viruses [49]. However, evidence indicates that the antigenicity of influenza viruses is determined by key amino acid substitutions around the receptor binding site. Koel et al. revealed that seven positions (i.e., 141, 151, 152, 154, 155, 185, and 189; H5 numbering) mainly determine the antigenicity of human seasonal H3N2 viruses [34]. Similarly, six positions, 129, 133, 151, 183, 185, and 189, were found to primarily influence the antigenicity of clade 2.1 H5N1 viruses [24]. In our study, positions 120, 126, 141, 156, 185, and 189 were recognized as key antigenic positions for clade 2.3.4.4 H5 viruses. Our findings further underscore that the antigenicity of influenza A viruses is mainly determined by key amino acids near the receptor binding site. Interestingly, all three studies independently identified positions 185 and 189, indicating that there may be a universal antigenic epitope among influenza A viruses.

Most reported neutralizing antibodies recognize conformational epitopes, like flu-20, 2D1, and HNIgGA6, but not linear epitopes [50,51,52]. As depicted in Figure 4, mAbs 1F1 and 2B5 recognized mutations at three positions (115, 120, and 126), suggesting that these three positions might form a conformational epitope. Mutations at all three positions may lead to a more pronounced antigenic change than a mutation at a single position. Similar observations were made for other positions; 156 and 185 likely form another conformational epitope recognized by mAb 3F6. Positions 156, 185, and 189 might form an epitope recognized by mAb 2C1. Positions 141, 185, and 189 might be part of a conformational epitope recognized by mAb 3B5. Additionally, positions 126, 141, and 156 might comprise a conformational epitope identified by mAb 1A10. Thus, our data offer insights into the identification of conformational epitopes. Since only eight mAbs were used in this study, comprehensive mapping of critical conformational epitopes influencing the antigenicity of influenza viruses using a broader array of mAbs is warranted. 

In this study, three positions (126, 156, and 189) were identified to be immunodominant, and two positions (126 and 156) were involved in glycosylation. Glycosylation in the HA stalk region plays a pivotal role in protein folding, trafficking, and pH stability. When located around the receptor binding site, glycosylation can either shield or reveal specific antigenic epitopes on HA proteins [32,40,53]. Human H3N2 viruses tend to acquire glycans to evade host immune responses. However, the gradual increase in glycosylation sites is not evident in H5 viruses (Figure 1). For clade 2.3.4.4 H5 viruses, glycosylation at residue 124N, resulting from an HA E126Δ mutation, has become dominant in natural H5N6 isolates [53]. These variants bypass the neutralizing activity of Re8-like serum antibody [53]. In contrast, the loss of glycosylation at residue 154N, resulting from the HA T156A mutation, has been observed in natural H5N6 isolates [54]. However, no significant difference in HI titers was detected between viruses with or without the T156A mutation [54]. In H7N9 viruses, the addition of glycosylation at residue 154N due to the HA A160T mutation has been observed, leading to a significant change in antigenicity [45]. The precise mechanism by which the T156A mutation leads to the loss of glycosylation at residue 154N, and its subsequent impact on antigenicity, requires further investigation. For position 189, Koel et al. found that the introduction of HA R189K was sufficient to change the antigenic properties of A/Indonesia/5/05 to become A/Chicken/North Sumatra/72/10-like viruses [24]. Thus, our findings from the antibody studies in vitro are consistent with the antigenic change that occurs in nature. Although these positions are immunodominant to induce host antibodies, their effects on the antigenicity of H5 viruses, when combined with substitutions in other positions, remain to be investigated.

Since 2014, H5 highly pathogenic avian influenza viruses bearing the clade 2.3.4.4 HA have spread among poultry and wild birds worldwide [4,21,22]. The persistent circulation of these HPAIVs in global poultry has given rise to eight distinct subclades (2.3.4.4a–h), resulting in different antigenic groups [23,38,55]. Our study underscores the role of specific antigenic positions in shaping the antigenic evolution of H5 viruses. Moreover, our findings provide valuable insights that can inform efforts to monitor and detect the emergence of new antigenic variants.

## Figures and Tables

**Table 1:** Hemagglutination inhibition antibody titers of mutant viruses with different antisera.

No.	Mutant	Antigenic Region	Sera			
			Re8	Re11	Re13	Re14
1	H5-Re8_L115Q	A	256	64	16	128
2	H5-Re8_K119R		256	64	16	128
3	H5-Re8_S120R		256	64	8	128
4	H5-Re8_P123S		256	32	8	128
5	H5-Re8_D124N/D126Δ *		64	32	8	64
6	H5-Re8_L129S		256	64	16	128
7	H5-Re8_M140V		256	64	16	128
8	H5-Re8_P141A		256	32	8	128
9	H5-Re8_I151T	B	256	64	8	128
10	H5-Re8_T156A		256	32	4	128
11	H5-Re8_N183S		256	32	8	128
12	H5-Re8_A185E		256	64	8	128
13	H5-Re8_T188I		256	64	16	128
14	H5-Re8_N189D		128	32	8	128
15	H5-Re8_R40K	C	256	32	8	128
16	H5-Re8_M269I		256	32	8	128
17	H5-Re8_H273N		256	32	8	128
18	H5-Re8_V198I	D	256	64	16	128
19	H5-Re8_V210A		256	64	16	128
20	H5-Re8_R72S	E	256	64	16	128
21	H5-Re8_A83T		256	64	16	128
22	H5-Re8_V257I		256	32	8	128
23	H5-Re8_K259T		256	32	8	128
24	H5_Re8	N.A.	256	32	16	128

Δ Amino acid deletion. * An additional mutation at this position introduced with D126Δ creates a potential glycosylation site. N.A., not applicable.

**Table 2:** Information about the monoclonal antibodies.

Name	Isotype(L)	HI Titer ^a^	MN Titer ^a^
2C1	IgM(κ)	256	+
1A10	IgG2a(κ)	256	+
2E12	IgG2a(κ)	256	+
2B5	IgG2b(κ)	256	+
3F6	IgG2b(κ)	128	+
3B11	IgG2b(κ)	256	+
1F1	IgG2b(κ)	256	+
3B5	IgG2b(κ)	256	+

^a^ Hemagglutination inhibition and microneutralization titer measure against the H5-Re8 vaccine virus; HI, hemagglutination inhibition; MN, microneutralization titer.

## Data Availability

Research data are available from Y.Z. and H.K upon request.
